# Retroperitoneal mesenchymal chondrosarcoma with metastasis to iliac vein: A rare case report and review of the literature

**DOI:** 10.1002/ccr3.6633

**Published:** 2022-11-23

**Authors:** Mahsa Masjedi Esfahani, Seyed Mohammad Ali Mirazimi, Javid Azadbakht, Fatemeh Dashti

**Affiliations:** ^1^ Department of Diagnostic Radiology, Kashan School of Medicine Kashan University of Medical Sciences Kashan Iran

**Keywords:** extraskeletal mesenchymal chondrosarcoma, iliac vein, magnetic resonance imaging, metastasis

## Abstract

The iliac vein is an extremely rare site of metastasis for extraskeletal mesenchymal chondrosarcoma (ESMC). Involvement of the veins usually leads to an extremely dismal prognosis. Here, we report a 50‐year‐old patient with retroperitoneal mesenchymal chondrosarcoma and initial metastasis to the iliac bone, which further progressed to involve the iliac vein. In this study, we reviewed the major characteristics of ESMC and the previously reported cases, considering the rarity of these tumors.

## Graphical abstract

Extraskeletal mesenchymal chondrosarcoma, a rare malignant neoplasm, has high local and metastatic recurrence rate and poor survival. We report here a rare case of iliac vein metastasis in ESMC. Surgical removal of the lesion remains the “gold standard” of treatment for localized tumors. For unresectable, advanced lesions, palliative chemotherapy is recommended.

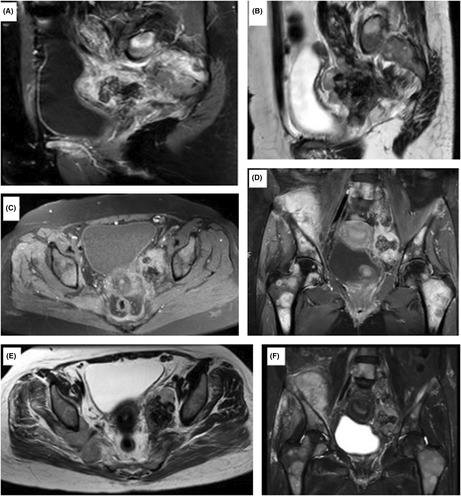

## 1 INTRODUCTION

Chondrosarcoma (CS) is a heterogeneous type of neoplasm which is specified by cartilaginous matrix production by the tumor cells. Chondrosarcoma is the third most prevalent bone malignancy after myeloma and osteosarcoma.^1^ Different types of CS have been pathologically identified, such as mesenchymal, conventional, dedifferentiated, myxoid, and clear cell.^2^ Mesenchymal chondrosarcoma (MCS) is a high‐grade type of CS that was evaluated for the first time in 1959 by Bernstein and Lichtenstein in 1959.^3^ The MCS is a rare and aggressive type of conventional chondrosarcoma that accounts for nearly 1% of all CSs and has a poor prognosis of 45%–55% survival in 5 years.^4^ 30% to 50% of MCSs have an extraskeletal origin.^5^ Most cases are diagnosed in the second decade of human life without any significant sex predilection.^6^ In this case report, we present a case of ESMC with metastasis to the left iliac vein, which to the best of our knowledge, has not been reported before.

## 2 CASE PRESENTATION

A 50‐year‐old woman with previously diagnosed retroperitoneal mesenchymal chondrosarcoma was referred to our hospital with the chief complaint of progressive left leg pain and swelling and paresthesia. The diagnosis of ESMC was made elsewhere during workup evaluation for sudden onset lower extremity deep vein thrombosis (DVT) with MRI findings of multiple T1, hypointense tissue lesions in the sacrum, femur and pelvic bones involving the bilateral iliac and pubic bones, left ischium with extension into the right acetabulum, right femoral head and ipsilateral lesser trochanter as well as proximal shaft of the femur consistent with chondrosarcoma. Histologic examination of the lesion biopsy was in favor of ESMC. The patient received five cycles of doxorubicin + ifosfamide regimen and was advised for surgical removal of the lesion, which was unsuccessful due to poor treatment compliance after discharge. Six months after the termination of last chemotherapy session, she referred to another hospital due to worsening of low back pain. Pelvic and lumbosacral MRI with Gadolinium revealed previous lesions along with evidence of bony metastasis involving the L3, L4, S1 and S2 vertebrae. This time, the patient was ordered four cycles of combined chemotherapy regimen (vincristine, cyclophosphamide, and doxorubicin) on Ewing's Sarcoma‐like protocol. During hospitalization, the patient developed COVID‐19 pneumonia and chemotherapy was discontinued for 2 months. Two months after hospital discharge, she referred to our hospital with progressive lower limb paresthesia and MRI evidence of enlarging previous lesions. Electromyography (EMG) and nerve conduction velocity (NCV) were performed to evaluate the cause of paresthesia, which showed early phase peripheral polyneuropathy due to chemotherapy. Considering the previous chemotherapy‐related adverse effects and lack of clinical response to earlier chemotherapeutic drugs, the patient received single‐agent gemcitabine regimen (1000 mg/sqm on day 1, 8, 15, every 28 days, administered intravenously in 30′). Clinical improvement was achieved as resolution of low back pain and paresthesia. However, four months later, she referred to our emergency department due to progressive constant low back pain unamenable to various analgesics. She also had limb edema, which started one week before her admission, and she lost her ability to walk on her side. On the physical examination, no ulceration or pigmentation was observed. No lymphadenopathy was found in the inguinal region. She has tender 4+ nonpitting edema on her left leg in conjunction with tactile tenderness of her lumbosacral region. Her right leg had limitations in mobility and movement. The ankle‐brachial index was 1.02 in the left leg and 1.04 in the right leg.

**FIGURE 1:**
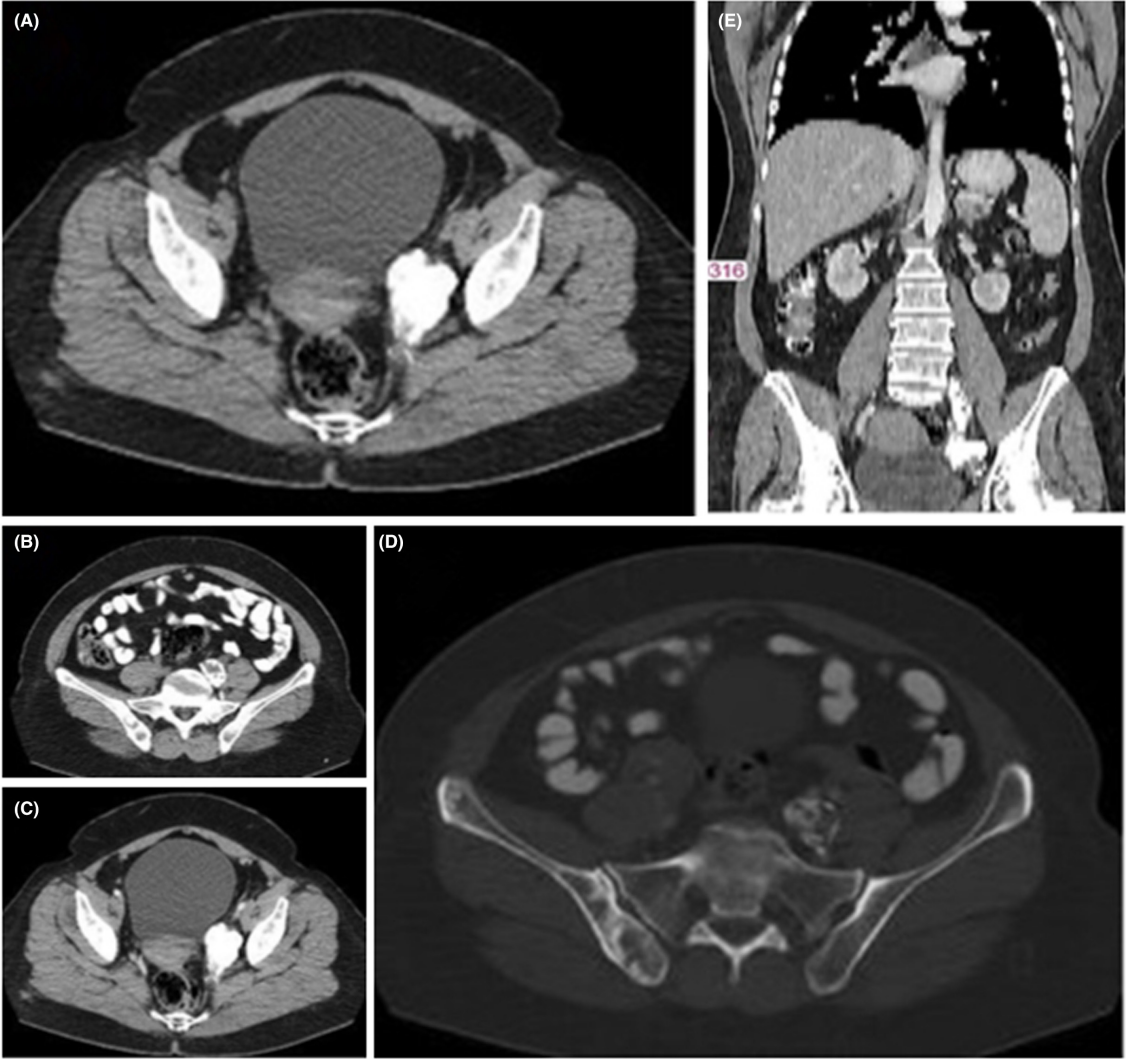
(A and B) noncontrast‐enhanced axial CT images exhibit a heterogeneous retroperitoneal mass with extensive dense popcorn‐like calcifications in the left paracervical area and the left common iliac vein. (C and D) Postcontrast images reveal only subtle heterogeneous enhancement of the mass mainly in its periphery. The lytic bony lesions are identified in the left iliac bone, indicative of metastasis. (E) Postcontrast coronal CT clearly displays the extension of the mass to the ipsilateral distended external, internal and common iliac veins.

**FIGURE 2:**
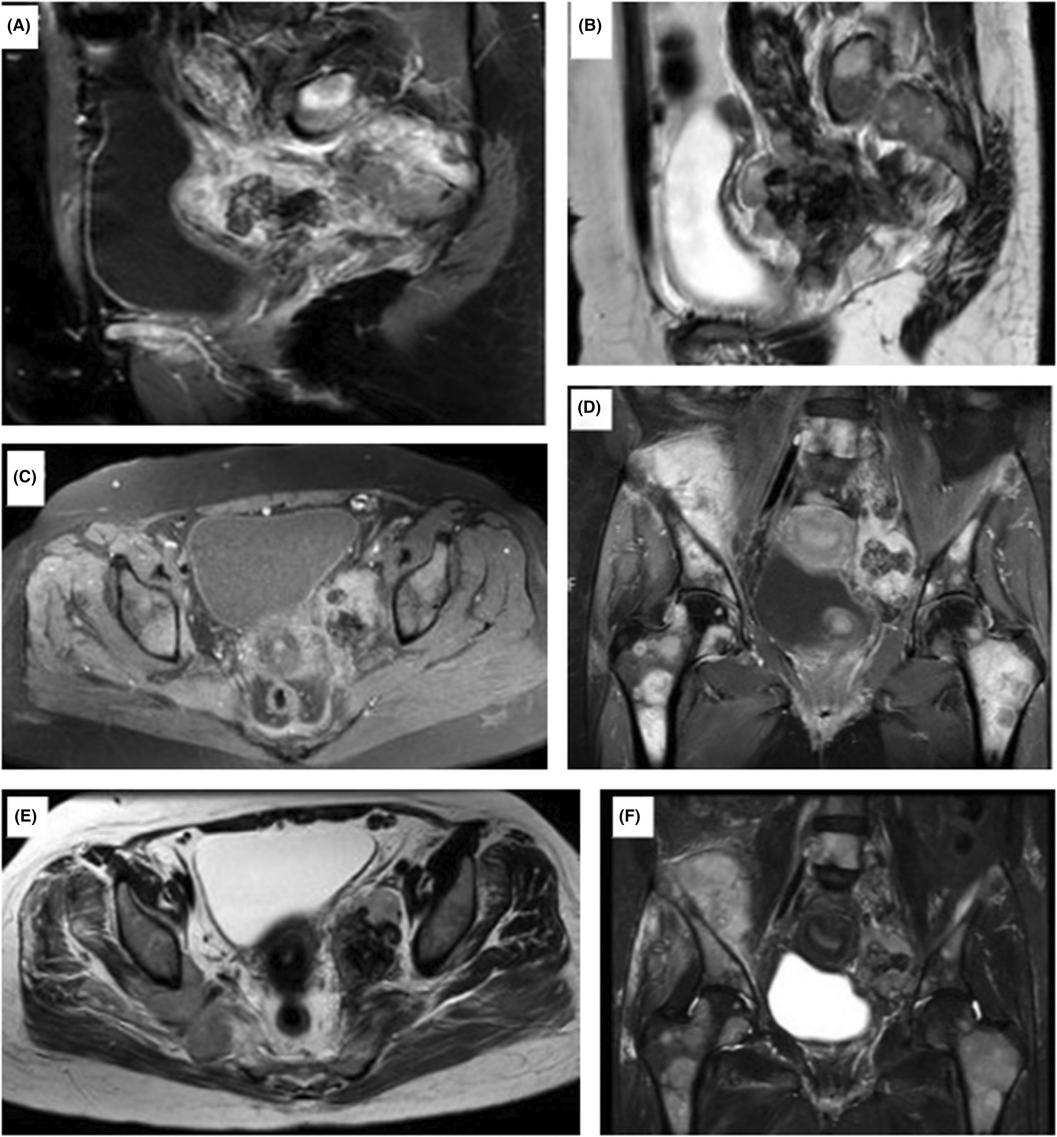
Coronal (A) and sagittal (C) contrast‐enhanced images revealed peripheral moderate enhancement of the non‐calcified components of the mentioned retroperitoneal mass and its extension to the left iliac vein complex as well as multiple enhancing bony metastases. The coronal STIR (B) and sagittal T2‐weighted (E) images can well display the extension of the mass to the expanded ipsilateral internal, external and common iliac veins. Axial T2‐weighted (F) and axial precontrast T1‐weighted (D) MR images represent a lobulated heterogeneous left paracervical mass with central popcorn signal voids (calcifications).

**FIGURE 3:**
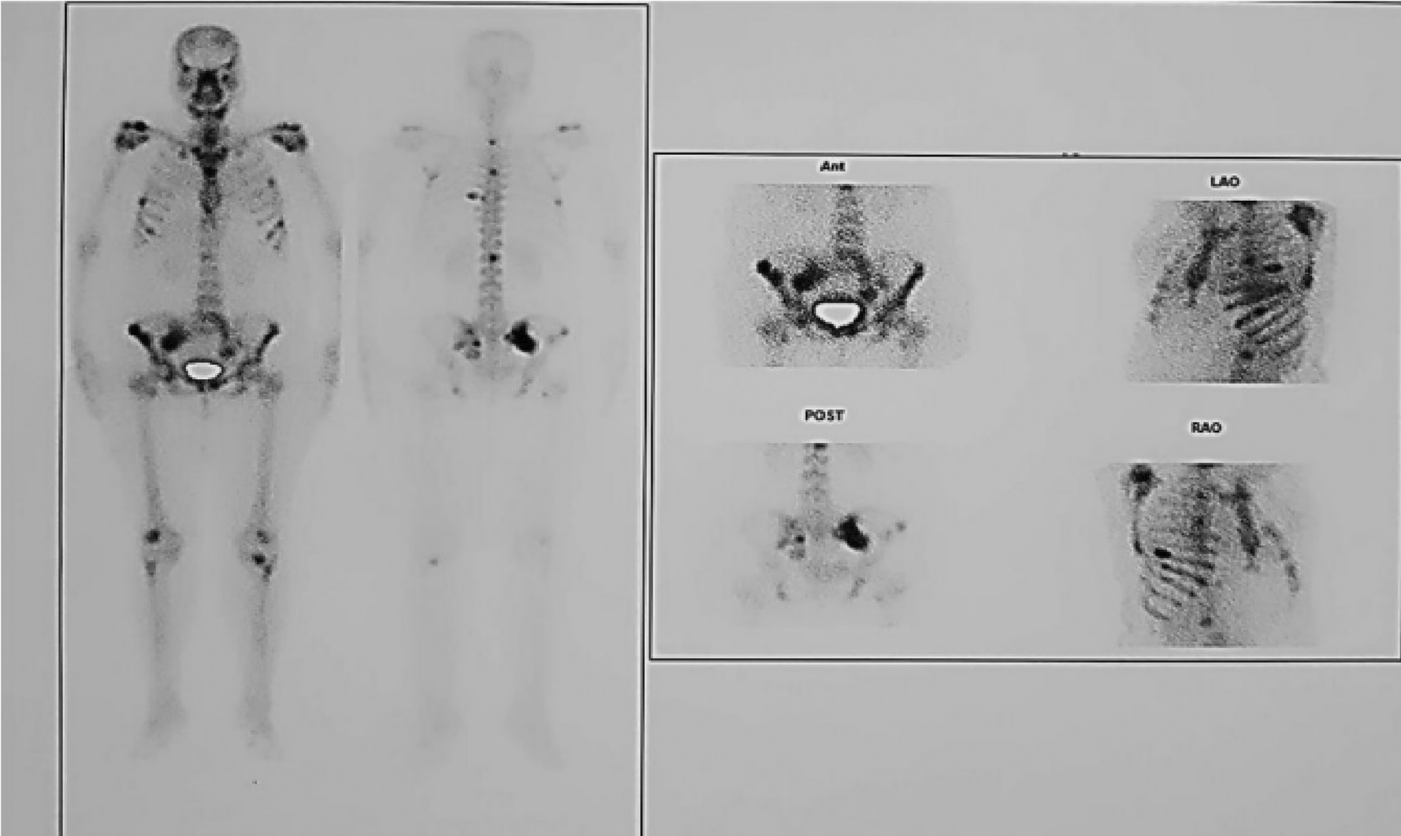
Bone scan represents multiple metastatic lesions involving bilateral pelvic bones, both femoral heads, some of the bilateral ribs as well as vertebral bodies.

The computed tomography (CT) images (Figure 1) displayed a minimally enhancing heterogeneous retroperitoneal mass with extensive dense popcorn‐like calcifications located in the left paracervical area extending to the ipsilateral distended external, internal and common iliac veins. Magnetic resonance imaging (MRI) with and without gadolinium contrast media (Figure 2) revealed a large lobulated hetrosignal lesion with internal popcorn signal voids (calcifications) in the left paracervical area invading to proximal of the left external/internal iliac veins and the distal portion of the left common iliac vein. Both MRI and bone scan (Figure 3) demonstrate multiple bone metastasis in pelvic and both femoral bones, with largest deposit in right iliac wing, breaking through internal and external cortices and expanding subperiosteally with aggressive and massive periosteal reaction. According to the MRI report, the tumor developed metastasis to the iliac vein, a rare location for mesenchymal chondrosarcoma to metastasis. Gemcitabine monotherapy was continued due to patient lack of consent for initiation of new drug regimen. She was discharged with partial clinical improvement. Six months later, she died due to sepsis and advanced disease.

## 3 DISCUSSION

Extraskeletal mesenchymal chondrosarcoma (ESMC) comprises a rare type of sarcoma originating from soft tissues, more frequently the meninges of the cranial and spinal cord, extremities, orbit and the lower extremities, particularly the thighs. Rarely, this tumor may arise from the retroperitoneum, kidneys, pancreas and hand musculature.^7^ Extraskeletal mesenchymal chondrosarcoma account for around 1% of all chondrosarcomas.^8^ Females are slightly more affected than males, while males carry a higher preponderance for extraskeletal and skeletal conventional chondrosarcomas.^7^ Extraskeletal mesenchymal chondrosarcoma has two peak ages of incidence: ESMC patients with involvement of the central nervous system, are generally younger (23.5 years old, range of 5–48 years old), while soft tissue and/or muscle tumor occur in older individuals (43.9 years old, range of 21–62 years old).^9^ Affected patients usually have unfavorable prognoses due to high rates of regional and distant metastasis.^10^ Surgical resection with wide local excision is considered as the treatment of choice for ESMC.^10^ Radiation therapy may be beneficial in patients with positive surgical margins as well as those who are not appropriate candidate for complete surgical removal of the tumor as it may lower the recurrence rate.^11^ Individuals with a more malignant ESMC, may benefit the most from the addition of adjuvant chemotherapy. Several cases of ESMC have been previously reported to show tumor remission in response to combined chemotherapy and radiotherapy.^10^ Several variable adjuvant chemotherapy regimens were recommended for the treatment of ESMC, including doxorubicin, dactinomycin, carbiplatin, cisplatin, etoposide, cyclophosphamide, vincristine and ifosfomide, of which doxorubicin has shown to be be crucial in all treatment regimens.^12^ Tumors with a high number of undifferentiated small cells with scant cartilage, were shown to be the most sensitive to chemo‐ and radiotherapy; however, it confers a less favorable prognosis and a more aggressive behavior.^10^ Chemotherap should be reserved in those with unresectable, advanced disease as a palliative treatment or as an adjuvant therapy in patients undergoing surgery and/or radiotherapy.^13^ In addition, patients with distant metastasis should receive individualized treatment. For example, young patients with limited distant metastasis may benefit the most from combined chemotherpay+ surgery or radiotherapy, whereas elderly patients with advanced disease are appropriate candidate for palliative chemotherapy.^14^ Our patient had variable chemotherapy regimens which were ultimately discontinued due to adverse effects and lack of response. A gemcitabine‐based single monotherapy was continued and led to partial symptom resolution. Gemcitabine may be considered as monotherapy in some cases of chondrosarcoma who fail to respond to multiple‐drug regimens.^15^ Collectively, there is no consensus regarding the chemotherapy regimen for the treatment of ESMC, with the exception of doxorubicin as the essential drug, and the majority of patients experience tumor relapse with distant metastases.

Retroperitoneal mesenchymal chondrosarcoma has been reported rarely in the literature. In a cross‐series study of Ghafoor et al.^16^ evaluating skeletal and extraskeletal mesenchymal chondrosarcoma imaging features, only 4% of cases were located in the retroperitoneum. ESMC can rarely involve the veins, with few cases being reported in the literature being as the primary site of the tumor. The iliac vein is an extremely uncommon site for ESMC and patients with primary iliac vein mesenchymal chondrosarcoma have a very poor prognosis.^17^ The femoral vein may also be the originating site for ESMC and it manifests with lower limb swelling and deep vein thrombosis.^18^ Moreover, intravascular mesenchymal chondrosarcoma may also arise from the pulmonary veins.^19^

Extraskeletal mesenchymal chondrosarcoma displays a strong tendency to locally and distantly metastasize, which makes the clinical outcome extremely dismal, with a reported 10‐year survival rate of 7%–26%.^20^ In a study by Frezza et al.^6^ conducted on 113 patients with ESMC, 17 patients (15%) presented with distant metastasis at the time of diagnosis: seven patients (42%) had pulmonary metastasis, two patients (11%) showed bone metastasis and eight patients (47%) had metastasis to multiple organs. Other previously reported sites of metastasis include lymph nodes^5^ and the scalp,^21^ adrenal glands, pancreas and kidneys.^16^ A review of the literature revealed one case of retroperitoneal mesenchymal chondrosarcoma metastasizing to the vein. Juan Hu et al.^9^ presented a 61‐year‐old woman with unintentional weight loss, persistent abdominal pain and nausea. Ultrasonography of the mass revealed two large retroperitoneal masses located adjacent to the inferior vena cava. The computed tomography scan showed dense and extensive, arc‐ and ring‐like calcifications in the retroperitoneal soft tissue mass. Abdominal and pelvic magnetic resonance imaging (MRI) with gadolinium enhancement was also performed, which showed hypointensity on T1‐weighted images and hyperintensity on T2‐weighted images associated with peripheral speculated enhancement consistent with calcification. Subsequently, the histologic examination of the lesions revealed ESMC. Our case is unique in that it was previously diagnosed with retroperitoneal mesenchymal chondrosarcoma with initial bone metastasis, which further progressed to involve the iliac vein (Table 1).

**TABLE 1:** Summary of cases of mesenchymal chondrosarcoma with involvement of the vein

Study	Age (years old)	Gender	Initial site of involvement	Site of metastasis	Radiologic features of the initial lesion	Reference
Sabharwal et al.	30 years old	male	right femoral vein	left pulmonary artery	‐	^18^
Zhang et al.	45 years old	female	Left iliac vein	Local invasion beyond the wall of the vein	Contrast‐enhanced computed tomography: a large lobulated mass in the left iliac vein with scattered calcifications	^17^
Simon et al.	25 years old	female	Left iliac vein	Paravertebral soft tissue	computed tomography scan: heterogeneous lesion with areas of granular, fine calcification T2WI: heterogeneous with fine hypointense foci due to calcification	^23^
Guo et al.	40 years old	male	Right femoral vein	Pancreas‐right upper pulmonary vein‐ right pulmonary hilum‐mediastinal and axillary lymph nodes‐ lung nodules	Computed tomography scan: Lesion with scattered calcification	^24^
Kim et al.	28 years old	female	Left femoral vein	No metastasis or local invasion	Computed tomography scan: multilobulated mass with scattered calcification	^25^
Oh et al.	41 years old	male	pancreas	splenic vein	Computed tomography scan: lobulated, heterogeneously enhancing necrotic mass with numerous areas of coarse calcification	^7^
JUAN HU et al.	61 years old	female	retroperitoneum	Inferior vena cava	Ultrasound: heterogeneous retroperitoneal masses with increased areas of echogenicity associated with dense posterior shadowing Computed tomography scan: heterogeneous areas with diffuse, dense, ring‐ and arc‐like calcifications T1W1: hypointense lesions T2WI: elevated signal intensity	^9^

Magnetic resonance imaging of ESMC often demonstrates equal or low signal intensity on T1W1 and heterogeneous hyperintense lesions on T2WI, as the intramural noncalcified and calcified regions of EMCS tend to have high and low intensity on T2WI, respectively, they are usually visible as areas of high signal intensity around areas with low signal intensity or the characteristic “salt and pepper” appearance.^22^ Moreover, contrast‐enhanced scanning of the lesions may show a diffuse nodular or heterogeneous pattern of enhancement, with the noncalcified component showing more homogenous enhancement and the calcified component showing less pronounced and heterogeneous enhancement, which can be clearly separated from each other in approximately 30% of cases. Calcifications appear predominantly as chondroid type arc‐ and ring‐like pattern in the majority of patients. In addition, T2‐hyperintense lobules, frequently seen in chondroid lesions, may be seen in 35% of patients. Moreover, skeletal involvement is associated with cortical destruction and extension into the surrounding soft tissue with periosteal reaction in some lesions.^16^ In this study, the patient showed similar MRI findings.

## 4 CONCLUSION

The current study shows the first case of extraskeletal mesenchymal chondrosarcoma with metastatic involvement of the iliac vein. Extraskeletal mesenchymal chondrosarcoma represents a rare entity of highly aggressive tumors with a propensity to metastasize locally and distantly. Magnetic resonance imaging findings include hypointense lesions on T1W1 and heterogeneously hyperintense lesions on T2WI. Metastases are common and involvement of less commonly reported sites may also occur.

## AUTHOR CONTRIBUTIONS

**Mahsa Masjedi Esfahani:** Conceptualization; data curation; formal analysis; investigation; methodology; project administration; supervision; visualization. **Seyed Mohammad Ali Mirazimi:** Conceptualization; data curation; investigation; methodology; resources; software; writing – original draft; writing – review and editing. **Javid Azadbakht:** Data curation; formal analysis; resources; validation; visualization. **Fatemeh Dashti:** Conceptualization; data curation; formal analysis; investigation; methodology; resources; software; validation; writing – original draft; writing – review and editing.

## FUNDING INFORMATION

No funding or sponsorship was received for this study or publication of this article.

## CONFLICT OF INTEREST

None declared. This manuscript has not been submitted to, nor is under review at, another journal or other publishing venue. The authors have no affiliation with any organization with a direct or indirect financial interest in the subject matter discussed in the manuscript.

## ETHICAL APPROVAL

Informed written consent was obtained from the patient for publication of this report and any accompanying images.

## CONSENT

Written informed consent was obtained from the patient to publish this report in accordance with the journal's patient consent policy.

## Data Availability

The data that support the findings of this study are available from the corresponding author, upon reasonable request.
